# Spatio-temporal evolution and trend prediction of urban ecosystem service value based on CLUE-S and GM (1,1) compound model

**DOI:** 10.1007/s10661-023-11853-y

**Published:** 2023-10-09

**Authors:** Hu Feng, Xu Lei, Guo Yu, Zhang Changchun

**Affiliations:** Department of Land and Resources, Hebei Agricultural University, Baoding 071000 Heibei, China

**Keywords:** Ecosystem service value, Land use, Dynamic evaluation, CLUE-S model, GM (1,1) model, Shijiazhuang

## Abstract

Ecosystem service value (ESV) is a significant indicator related to regional ecological well-being. Evaluating ESV premised on continuous time series land benefit data can provide an accurate reference for regional ecological civilization construction and sustainable development. Taking Shijiazhuang, the capital city of Hebei Province as an example, the study analyzed land use changes based on the land use data of the continuous time series from 2000 to 2020 and introduced a socio-economic adjustment factor and biomass factor adjustment factor to construct a dynamic assessment model of ecosystem service value. The spatiotemporal changes of the ecosystem service value in Shijiazhuang City were evaluated, and the dynamic prediction of the ecosystem service value was made using the CLUE-S model and the GM (1,1) model. (1) The changes in the overall ESV and spatial pattern in Shijiazhuang are strongly linked to the change in land use, and the contribution of cultivated land, woodland, and grassland to ecosystem service value exceeds 90%. (2) Between 2000 and 2020, the value of ecosystem services illustrated a dynamic change and gradually declined, with the total amount falling from 28.003 to 19.513 billion yuan. Among individual ecosystem services, the value of regulation services suffered the most serious loss. (3) CLUE-S and GM (1,1) perform well in the prediction of ESV. The prediction outcomes illustrate that the ecosystem service value of Shijiazhuang will continue to decline by 2025, and the ecosystem value will drop to 16.771 billion yuan. This research may offer a reference for the dynamic assessment of ESV of the continuous sequence and help to promote regional ecological protection and sustainable development.

## Introduction

Ecosystem services are a general term for products or services that can be directly or indirectly provided to human beings in the structure, function, or process of the ecosystem (Costanza et al., 2014; Daily et al., 2000), such as providing raw materials, purifying the environment, and maintaining biological properties (Jing et al., 2021), which is an essential foundation for ensuring the stability as well as sustainable development of the regional ecological environment (Reid et al., 2005; Xi et al., 2021). However, due to human activities such as industrialization (Opoku & Aluko, 2021), rapid population expansion (Dimnwobi et al., 2021), and excessive consumption of resources (Mittal & Gupta, 2015), global ecosystem services have been seriously damaged (Chen et al., 2019; Costanza et al., 2014). In 2005, the landmark United Nations Millennium Ecosystem Assessment Report was released (Vihervaara et al., 2010), revealing the historical facts of ecosystem service degradation and clearly pointing out the important role of ecosystem service assessment in ecological protection (Xu et al., 2020), decision optimization (Mirghaed et al., 2020), resource utilization (Costanza et al., 2014), and so on. Quantitative assessment of ecosystem services is a significant method to assess ecosystem services, which can further characterize, simulate, and monitor ecosystem services (Carpenter et al., 2009), and has gradually become a predominant issue in the field of ecology (Costanza et al., 2017; Xi et al., 2021).

Currently, the commonly used methods of quantitative ecosystem service evaluation include material quality assessment, energy value assessment, and value quantity assessment (Yuan et al., 2019a, b), in which the value quantity assessment method based on market theory is to quantitatively assess the services provided by ecosystems from the perspective of monetary value quantity, monetize the value of ecosystem services, and performs an important function in raising people’s awareness of environmental protection, promoting environmental changes to be incorporated into the national economic accounting system, and formulating economic incentive measures (Luisetti et al., 2013). Since Costanza et al. put forward and used this method in 1997 to comprehensively evaluate the value of 17 kinds of ecosystem services in the world (Costanza et al., 1997), scholars have used or improved this method to compute the ecosystem service values (ESV) (Costanza et al., 2014; Zhang et al., 2022) and obtained a series of research results (Yu, 2021). In China, Xie et al. (2008), based on the research of Costanza et al. (1997), through expert consultation and a willingness survey of more than 700 ecologists, constructed the “Equivalent Scale of China's Terrestrial Ecosystem Services Value,” which was consistent with China’s regional characteristics and the actual development, and achieved a large number of applications (Pan et al., 2021; Wu et al., 2020; Xue & Ma, 2018). Specifically, scholars have extensively analyzed the spatial and temporal distributions and the drivers of ESV based on the ecosystem service value equivalence scale at the national (Yuan et al., 2019a, b), watershed (Hou et al., 2021), and provincial scales (Zhang et al., 2020a), and explored the impacts of human activities on ESV by combining the concepts of landscape pattern, ecological compensation, and ecological risk.

Land use/cover change is the direct embodiment of the interaction between human activities and environmental changes (Costanza et al., 2014). Changes in land use structure, intensity, and scale will influence the structure and function of the regional ecological environment to a certain extent (Gaglio et al., 2017; Hasan et al., 2020), and as a consequence, the provision of regional ecosystem services is impacted (Liu et al., 2020; Xi et al., 2021). Cities are experiencing the most significant land-use changes globally, with rapid urbanization adversely affecting the structure and function of urban ecosystems and leading to significant alterations in the ESV (Yu et al., 2022). In order to further uncover the spatial and temporal responses of urban ESV to land use changes, as well as the mechanisms by which land use changes impact urban ESV, scholars have undertaken numerous empirical studies, including the spatial migration of ESV (Liu et al., 2021a), the spatially uneven response of ESV to urbanization (Li et al., 2021a, b), and scenario simulations of ESV (Lou et al., 2022). However, most current research focuses solely on static evaluations of ESV from the land use perspective. There’s an absence of consideration for the dynamic shifts in natural, economic, social, and other factors, which makes it challenging to depict the evolving nature of ESV influenced by a combination of these elements.

Depending on the characteristics of land use in historical periods, predicting the future land use demand and evaluating the ESV are related to the future ecological well-being of the region (Yirsaw et al., 2017). In practice, models such as the conversion of land use and its effect at small regional extent (CLUE-S) (Peng et al., 2021), future land use simulation (FLUS) (Li et al., 2020), and patch-generating land use change simulation model (PLUS) (Lou et al., 2022) are used to predict land use changes and explore the spatiotemporal variations of ESV. The results of these models are actively incorporated into local management decisions (Liang et al., 2017; Xing et al., 2020). Among them, the CLUE-S model can quantitatively evaluate the influence of natural, social, and economic factors on land use change based on a bottom-up perspective, and at the same time, it can allocate land use demand from top-down. It has obvious advantages in small-scale land use refinement simulation (Wu et al., 2020) and can accurately depict the future land use pattern, providing accurate data support for developing regional future ecosystem service value. However, most current studies estimate future ESV based on land use prediction data and the existing regional natural environment and socio-economic development level, lacking consideration for the dynamic changes of the natural environment and social and economic development levels. Consequently, the study made a bold attempt to predict the indicators indicating the degree of economic and social development, such as per capita gross domestic product (GDP) and the proportion of the urban population, and apply them to the future evaluation of the ESV.

ESV is the result of many factors’ mutual influence and interaction (Liu et al., 2021b), and land use change is the main factor (Li et al., 2018; Zhang et al., 2020a). However, due to regional differences such as climate conditions and socio-economic development levels, ESV shows obvious spatial and temporal heterogeneities (Wang et al., 2016). Therefore, with the Shijiazhuang region of China serving as an illustration and predicated on the land use data of long-term time series data from 2000 to 2020, this paper introduced the social and economic adjustment coefficients and the biomass factor adjustment coefficients to construct a dynamic evaluation model of ESV, and explored the spatiotemporal evolution characteristics of ecological system service value and uses CLUE-S model and GM (1,1) model to predict its dynamic changes. The aim is to explore the dynamic evolution characteristics of ESV from a continuous time-series perspective, fill the corresponding research gaps, and provide a theoretical basis and scientific reference for regional ecological environment protection and sustainable development.

## Materials and methods

### Research area

**Fig. 1:**
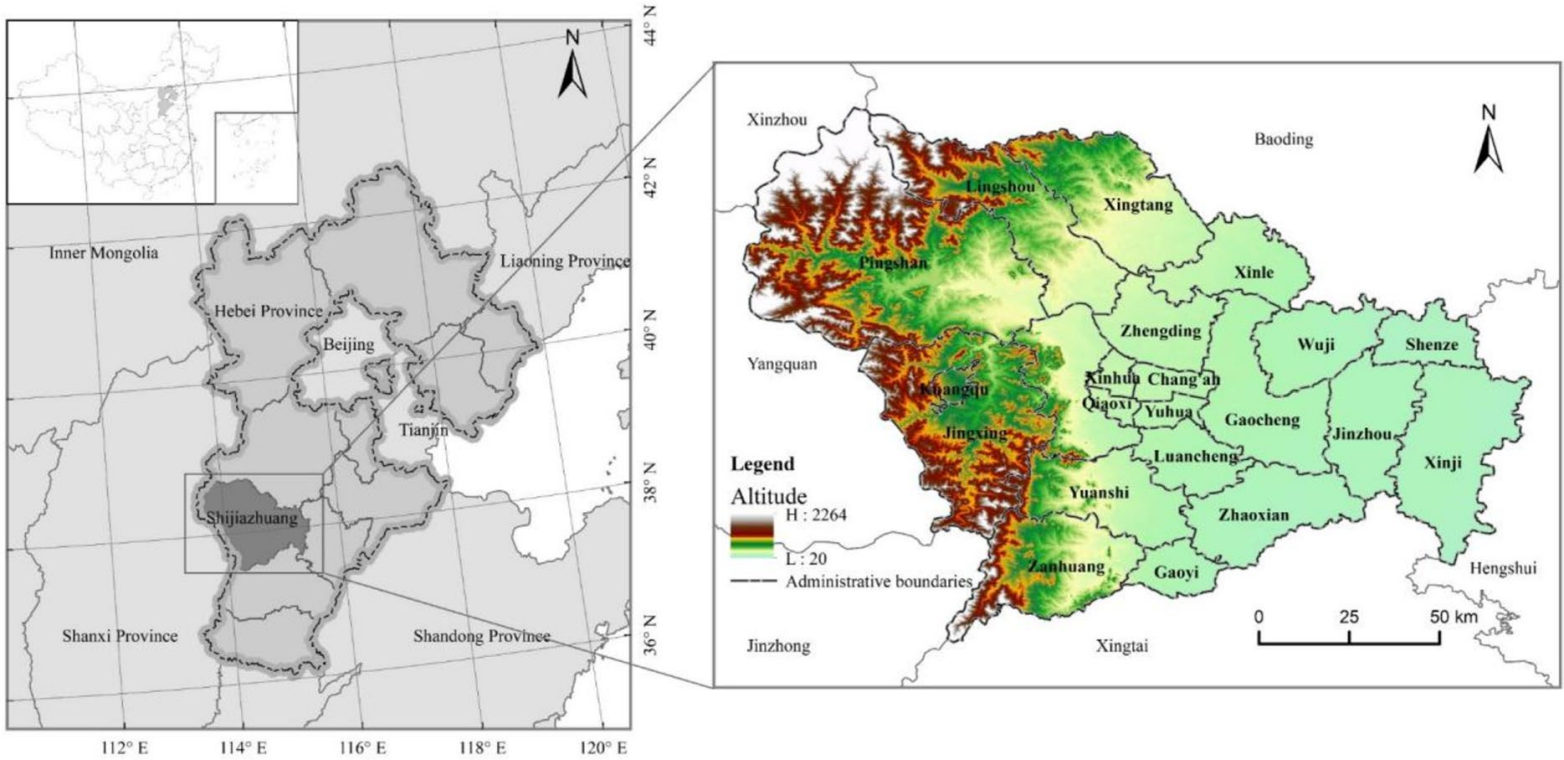
Location map of the study area

Shijiazhuang (37°27′–38°47′N, 113°30′–115°20′E) is located in the south-central part of Hebei Province, bordering Baoding and Hengshui in the east, Xingtai in the south, and Xinzhou, Yangquan, and Jinzhong in Shanxi Province in the west (Fig. 1). The terrain is low in the east and high in the west, spanning two geomorphological units of Taihang Mountain and North China Plain, and the middle part of Taihang Mountain in the west. The terrain is dominated by mountains, and the east is a vast plain area. The climate is temperate continental monsoon, with four distinct seasons, simultaneous heat and rain, an annual average temperature of 12.2–13.2 ℃, annual rainfall of 477.45–68.8 mm, and abundant animal and plant resources.

Shijiazhuang is the cultural, economic, and political center of Hebei Province. It has authority over a total land area of 14,058.5 km^2^ and encompasses 8 districts, 11 counties, and 3 cities located at the county level. Shijiazhuang has witnessed remarkable achievements in economic development. By the end of 2020, its permanent population reached 10.641 million, and the GDP amounted to 593.51 billion yuan. The urbanization rate stood at 70.6%, marking an increase of 33.9% from 2000. However, there has also been a continual expansion in construction land. By the end of 2020, the urban built-up area expanded to 338.2 km2, leading to significant alterations in the land use structure and considerable encroachment on ecological land space. As a representation of the development trajectory of many Chinese cities, Shijiazhuang, against the backdrop of swift urbanization and industrialization, has encountered various ecological and environmental challenges, including food security issues, water shortages, and air pollution. The regional ecosystem services and ecological safety are under grave threats. Hence, using Shijiazhuang as a case study, research has been undertaken on the ESV. The goal is to decipher the dynamics of urban ESV, providing scientific insights to address escalating human-land conflicts and to refine ecosystem service management in the face of rapid urban expansion.

### Data collection and processing

The basic data of continuous land use between 2000 and 2020 are from the Annual China Land Cover Dataset (CLCD) (10.5281/zenodo.4417810), with a spatial resolution of 30 × 30 m (Yang & Huang, 2021). The evaluation shows that the accuracy of this data is better than that of MCD12Q1, GlobeLand30, FROM_GLC, and other land use classification data, and it has obvious advantages in reflecting the changes in the land use system as a direct result of human activities (Yang & Huang, 2021). According to the research needs, we re-divided and integrated the initial land use types and categorized them into unused land, cultivated land, construction land, water area, grassland, and woodland.

The continuous sequence temperature and rainfall data are all from China’s 1 km resolution monthly rainfall data set and 1 km resolution monthly average temperature data set (http://data.tpdc.ac.cn/zh-hans/) published by China International Qinghai-Tibet Plateau Scientific Data Center (Ding & Peng, 2020; Peng et al., 2019). Furthermore, to better meet China’s ecological civilization construction strategy and “double carbon” construction goal, we use the estimated temperature and rainfall data under the SSP119 scenario (O’Neill et al., 2016) as the basic data in the stage of ecosystem service value prediction (Peng et al., 2017). Social data come from the Statistical Bulletin of China’s National Economic and Social Development (2001–2021), China Statistical Yearbook (2001–2021), Shijiazhuang Statistical Yearbook (2001–2021), and Shijiazhuang Government Work Report (2001–2021).

The CLUE-S model is used for land use forecasting and modeling. The elevation data used are from ASTER Global Digital Elevation Database (https://asterweb.jpl.nasa.gov/GDEM.asp) with a spatial resolution of 30 × 30 m. The road network data comes from the open block (https://www.openstreetmap.org); distribution data of government stations, railways, and rivers are from China National Basic Geographic Information Center (http://www.ngcc.cn/ngcc); GDP and NDVI index are from the Resource and Environmental Science and Data Center of Chinese Academy of Sciences (https://www.resdc.cn/) with a spatial resolution of 1 × 1 km; the WorldPop database (https://www.worldpop.org/) provided information about the population density with a spatial resolution of 100 × 100 m.

### Research methods

#### Evaluation model of ecosystem service value

The ESV may provide information on the structure and function of ecosystem services, which could be used to support or optimize the management decisions of regional sustainable, healthy, and stable development (Su et al., 2020). In 2003, Xie et al. (2003) first proposed a technique for assessing the value of terrestrial ecosystem services in China, and then revised it in 2008 to obtain the equivalent coefficient of ecosystem services and land types (Xie et al., 2008), and proved its robustness and feasibility through sensitivity test (Xue & Ma, 2018). According to related research (Xie et al., 2008), the ESV of one standard equivalent factor was shown to be identical to the economic value of the annual natural output of 1 hm^2^ farmland, and the value of one equivalent factor was roughly equal to 1/7 of the grain yield price (Su et al., 2020; Xue & Ma, 2018). In this case study, based on the regional heterogeneity of ESV (Su et al., 2020), the equivalent coefficient of ESV is recalculated to establish the ESV coefficient in line with the study area. The following is the formula for the computation:

1 $${E}_{n}=\frac{1}{7}\times \left(P\times Q\right)$$

whereby, $E_n$ denotes the economic value generated from the unit area of farmland in Shijiazhuang, and the unit is RMB/hm^2^; *P* refers to the average price of food crops in Shijiazhuang in 2020, 1.23 RMB/kg; *Q* denotes the average produce of main food crops in Shijiazhuang from 2000 to 2020, which is 6591.33 kg/hm^2^. Through calculation, the economic value of farmland ecosystem services for each unit area in Shijiazhuang is 1158.19 RMB/hm^2^, and then the ESV coefficient of Shijiazhuang is calculated (Table 1). Among them, the construction land is disturbed by human activities to a high degree. According to previous research viewpoints (Li et al., 2020), the ESV of the construction land is 0.

**Table 1:** Equivalent value of ecosystem services per unit area of Shijiazhuang ecosystem (RMB/hm^2^)

ESs		Land use category				
First category	Second category	Plowland	Forestland	Grassland	Water area	Unused land
Provisioning services	Food production	1158.19	382.20	498.02	613.84	23.16
	Raw material	451.69	3451.41	416.95	405.37	46.33
Regulating services	Gas regulation	833.90	5003.38	1737.29	590.68	69.49
	Climate regulation	1123.44	4713.83	1806.78	2385.87	150.56
	Hydrological regulation	1609.88	1992.09	1528.81	17,199.12	301.13
Supporting services	Waste treatment	891.81	4737.00	1760.45	21,739.23	81.07
	Soil formation and retention	1702.54	4655.92	2594.35	474.86	196.89
	Biodiversity protection	1181.35	5223.44	2165.82	3972.59	463.28
Cultural services	Recreation and culture	196.89	2409.04	1007.63	5142.36	277.97
Total		9149.70	32,568.30	13,516.08	52,523.92	1609.88

To further accurately evaluate the ESV, the study introduced biomass factors and socio-economic factors for revision and constructed a dynamic evaluation model of ESV that can reflect the actual features of the study area (Fei et al., 2018; Su et al., 2020). The formula is as follows:

2 $$ESV=\sum \left({A}_{k}\times V{C}_{k}\times {S}_{k}\times PI\right)$$

whereby, *ESV* represents the ecosystem service value (RMB); ${A}_{k}$ denotes the area of land use type *k* (hm^2^); $V{C}_{k}$ denotes the value coefficient, which represents the service value per unit area of land use type *k* (yuan/hm^2^); ${S}_{k}$ denotes the adjustment coefficient of biomass factor; *PI* denotes the adjustment coefficient of economic and social factors.

Among the above adjustment parameters, it is necessary to further revise the adjustment coefficient ${S}_{k}$ of biomass factor. Combining with the feasibility of data acquisition, the factor will be adjusted based on the results of a comparison of the net primary productivity (NPP) of vegetation in various areas. The formula is as follows:

3 $${S}_{k}=\frac{NP{P}_{s}}{NP{P}_{g}}$$

whereby, $NP{P}_{s}$ denotes the net primary productivity of vegetation in Shijiazhuang; $NP{P}_{g}$ denotes the net primary productivity of vegetation in China.

The net primary productivity of vegetation is often used to describe the distribution of natural vegetation productivity on a regional scale. The Thornthwaite Memoria model that was developed by Lieth and Box (1972) is utilized for computation, and it is based on the findings of the previous studies. The model formula is as follows:

4 $$\begin{aligned}NPP =&\; 3000\times \left[1-{e}^{-0.0009695\left(V-20\right)}\right]\\& V=\frac{1.05\;Pre}{\sqrt{1\mathop{+}{\left(1\mathop{+}1.05Pre/L\right)}^{2}}}\\& L=3000-25Tmp+0.05Tm{p}^{3}\end{aligned}$$

whereby, *NPP* denotes the net primary productivity of vegetation (t/hm^2^); *V* denotes the actual annual evapotranspiration (mm); *L* denotes the annual average evapotranspiration (mm); *Tmp* denotes the annual average temperature (℃); *Pre* denotes the average annual rainfall (mm).

Residents’ desire and capacity to pay are used in the calculation of the adjustment coefficient of social variables, and the formula is as follows:

5 $$PI={W}_{t}\times {A}_{t}$$

whereby, ${W}_{t}$ is the coefficient of people’s willingness to pay for ecosystem services, which may be determined by the use of the logistic regression model. The higher the value, the stronger the willingness to pay; ${A}_{t}$ represents the coefficient of people’s ability to pay for the value of ecosystem services, which may be computed by per capita GDP. The formula of ${W}_{t}$ is as follows:

6 $${W}_{t}=\frac{{W}_{s}}{{W}_{g}}$$

whereby, ${W}_{s}$ is the parameter of Shijiazhuang’s willingness to pay; ${W}_{g}$ is a parameter that measures the national willingness to pay. Among them, the willingness to pay parameters are calculated as follows:

7 $$\begin{aligned}W=&\;\frac{2}{\left(1\mathop{+}{ae}^{-bm}\right)}\\& m=\frac{1}{{E}_{nt}}-2.5\\& E{n}_{t}=E{n}_{tr}\times {P}_{tr}+E{n}_{tu}\times {P}_{tu}\end{aligned}$$

whereby, *W* is the calculation parameter of willingness to pay coefficient ${W}_{t}$; *m* is a measure of the social development stage’s coefficient, and its value is inversely proportional to the level of social development; *a, b* are both 1 for simplified calculation; $E{n}_{t}$ is Engel’s coefficient in the *t* year of the study area; $E{n}_{tr}$ and $E{n}_{tu}$, respectively, represent Engel’s coefficient of rural and urban areas in the *t* year; ${Pn}_{tr}$ and ${Pn}_{tu}$, respectively, represent the population proportion (%) of rural and urban areas in the *t* year.

The ability to pay coefficient $\left({A}_{t}\right)$ can be reflected by the per capita production capacity, and the formula is as follows:

8 $${A}_{t}=\frac{GD{P}_{mst}}{GD{P}_{mt}}$$

whereby, $GD{P}_{mst}$ represents the per capita GDP (RMB/person) of Shijiazhuang in the *t *year; $GD{P}_{mt}$ means the national per capita GDP (RMB/person) in the *t* year.

#### CLUE-S model

By using the original CLUE model as the foundation, Verburg et al. (2002) created the CLUE-S model, which is a land use change simulation model, which is widely applied in the research of land use pattern evolution and prediction (Han et al., 2015; Liao et al., 2022). The model is broken up into a non-spatial module and a spatial module, both of which are based on the guiding premise that the spatial pattern of land use is the outcome of the interplay of land use demand, the natural environment, and the social economy, and other influential elements. Non-spatial models are used to calculate land use demand, while spatial models allocate land use demand to specific locations. In this study, CLUE-S land use prediction modeling is completed through four parts, and the kappa coefficient is applied to the data to determine how accurate the model is. Details on the evaluation criteria and principles used in this study can be found in Verburg et al. (2002).

##### Forecast of land use demand

The acquired land use demand data is generated by the Markov model, which can predict the future land use change according to the transfer probability of the current land use change (Han et al., 2015). Firstly, the probability matrix of land use transfer is computed depending on the data of land use in 2015 and 2020, and the predicted amount of land use in 2025 is obtained predicated on the probability of land use transfer. Then, the amount of land use changes from 2020 to 2025 is allocated to each year by linear interpolation.

##### Calculation of distribution probability of land use

The spatial distribution of land use is affected by natural conditions, social economy, and policy environment. Therefore, referring to other people’s research findings and the actual conditions of the study area (Liao et al., 2022), the study selects 13 factors including elevation, population density, and GDP as adaptive variables influencing the spatial distribution of land use, and uses the logistic regression model to determine the influence degree of each factor. The equation is expressed as follows:

9 $$Log\left(\frac{P_i}{1-P_i}=\beta_{0,1}+\beta_1X_{1,i}+\beta_2X_{2,i}+\cdots\cdots+\beta_nX_{n,i}\right)$$

whereby, ${P}_{i}$ denotes the probability of land use type *i* appearing in the spatial grid; ${X}_{n}$ is the *n*-th influencing factor; ${\beta }_{\mathrm{0,1}}$ denotes the intercept; ${\beta }_{n,1}$ signifies the regression coefficient of the *n*-th factor of land type *i*. To ensure the reliability of the logistic regression equation, relative operating characteristics (ROC) are used to test the model’s accuracy (Verburg et al., 2002). Generally speaking, when the ROC value is closer to 1, the probability of land benefit distribution calculated by the regression model is closer to the real land-type distribution. From Table 2, the chosen driving factors of land use have strong explanatory power to the land use spatial distribution, and the regression equation to be adopted by the model is more reliable.

**Table 2:** Land use in Shijiazhuang as determined via binary Logistic regression

Driving factors	Plowland	Forestland	Grassland	Water area	Construction land	Unused land
Elevation	− 18.712	3.514	− 1.012	− 68.452	− 13.229	
Slope	− 9.722	5.246	2.789	− 15.775	− 6.276	9.016
Aspect	0.086	0.621	− 0.525	− 0.692	0.196	
GDP	− 2.961	− 38.827	− 12.377	− 1.898	1.424	
NDVI	11.793	9.081	− 6.729	− 7.644	− 11.883	− 12.180
Temperature	− 4.461	− 5.420	− 6.343	− 23.744	− 6.642	
Rainfall	− 3.717	− 7.575	− 2.211	16.960	0.043	6.329
Population density	− 40.272	− 477.199	− 470.841	− 0.880	15.585	
Distance to the river	1.840	− 0.228	1.345	− 3.773	− 0.473	
Distance to the highway	− 1.227	0.054	− 2.143	1.427	− 1.501	
Distance to the railway	1.244	2.279	2.219	5.579	− 0.235	− 7.914
Distance to the local road	− 1.227	0.872	0.129	16.926	− 15.335	6.226
Distance to administrative center	− 0.528	− 4.071	1.252	1.327	− 1.079	
Constant	0.000	− 1.079	7.405	31.098	14.777	− 6.372
ROC value	0.930	0.968	0.897	0.953	0.937	0.990

##### Flexible setting of land use conversion

The elasticity coefficient of land use conversion is between 0 and 1, with “0” indicating that land types are easy to be transferred, and “1” indicating that land use types are difficult to be transferred. As per the actual scenario of land use conversion from 2000 to 2020, after repeated fitting, screening, and adjustment, the conversion elasticity coefficients of 6 types of land use are finally set as cultivated land 0.75, woodland 0.85, grassland 0.80, construction land 0.85, water area 0.90, and unused land 0.70.

##### Setting the space constraint area

A spatial constraint is the spatial expression of national or local policies, which is of utmost importance to the maintenance of people’s livelihood development, social stability, and ecological security. Huangbizhuang Reservoir and Gangnan Reservoir bear the heavy responsibility of water supply, drought, and flood control in Shijiazhuang City. Since 1987, the Regulations on Prevention and Control of Drinking Water Source Pollution in Huangbizhuang Reservoir in Gangnan, Shijiazhuang City, have been issued, and the water area control and protection have been continuously carried out. At the same time, in recent years, Hebei Province is gradually promoting the greening construction of Taihang Mountain and Yanshan Mountain, and forest land protection is highly valued by Shijiazhuang. To sum up, the study set the Huangbizhuang Reservoir, Gangnan Reservoir, and the forest park area in Shijiazhuang to not transfer to other land types.

#### GM (1,1) model

GM (1,1) may differentiate between the various degrees of development trends shown by system factors, as well as produce and analyze the original data to derive the system evolution law, and then establish a gray model to forecast the system while taking into account undefinable variables (Pan et al., 2021), which is widely used in the evaluation and decision-making of economy (Yuan & Chen, 2016), geography (Wang & Cao, 2021a, b), ecology (Chen et al., 2020), and other fields. This study is used to forecast the trend of three indicators of socio-economic development level: GDP, Engel’s coefficient, and urban population ratio.

If ${x}^{\left(0\right)}\left(i\right)$ is used to represent the original sequence and ${x}^{\left(1\right)}\left(i\right)$ represent the first-order cumulative sequence, the standard formula of the first-order linear differential equation of GM (1,1) model will be

10 $$\frac{d{x}^{\left(1\right)}}{dt}+a{x}^{\left(1\right)}=u$$

The standard solution of GM (1,1) model is

11 $${x}^{\left(1\right)}=\left({x}^{\left(0\right)}\left(1\right)-\frac{u}{a}\right){e}^{-at}+\frac{u}{a}$$

whereby, *a* and *u* are metrics to be ascertained and *t* represents time. The posterior error test is a technique that is utilized to assess the accuracy of the model and was applied to verify the reliability as well as the accuracy of the GM (1,1) model prediction. The posterior error ratio *C* and the small error frequency *P* are defined as

12 $$\begin{array}{c}C=\frac{{S}_{2}}{{S}_{1}}\\ P=\left\{\left|{\varepsilon }_{K}-\overline{\varepsilon }\right|<0.6745\;{S}_{1}\right\}\end{array}$$

whereby, ${S}_{1}$ represents the variance of the initial data expressed as a standard deviation; ${S}_{1}$ denotes the standard deviation of predicted data; ${\varepsilon }_{K}$ denotes the prediction data error; $\overline{\varepsilon }$ is the average of prediction errors. When the model satisfies *P* > 0.95 and *C* < 0.35, the predictive accuracy level of the model is excellent, and it can be used for system prediction (Hu et al., 2020).

## Results and analysis

### Land use change

**Fig. 2:**
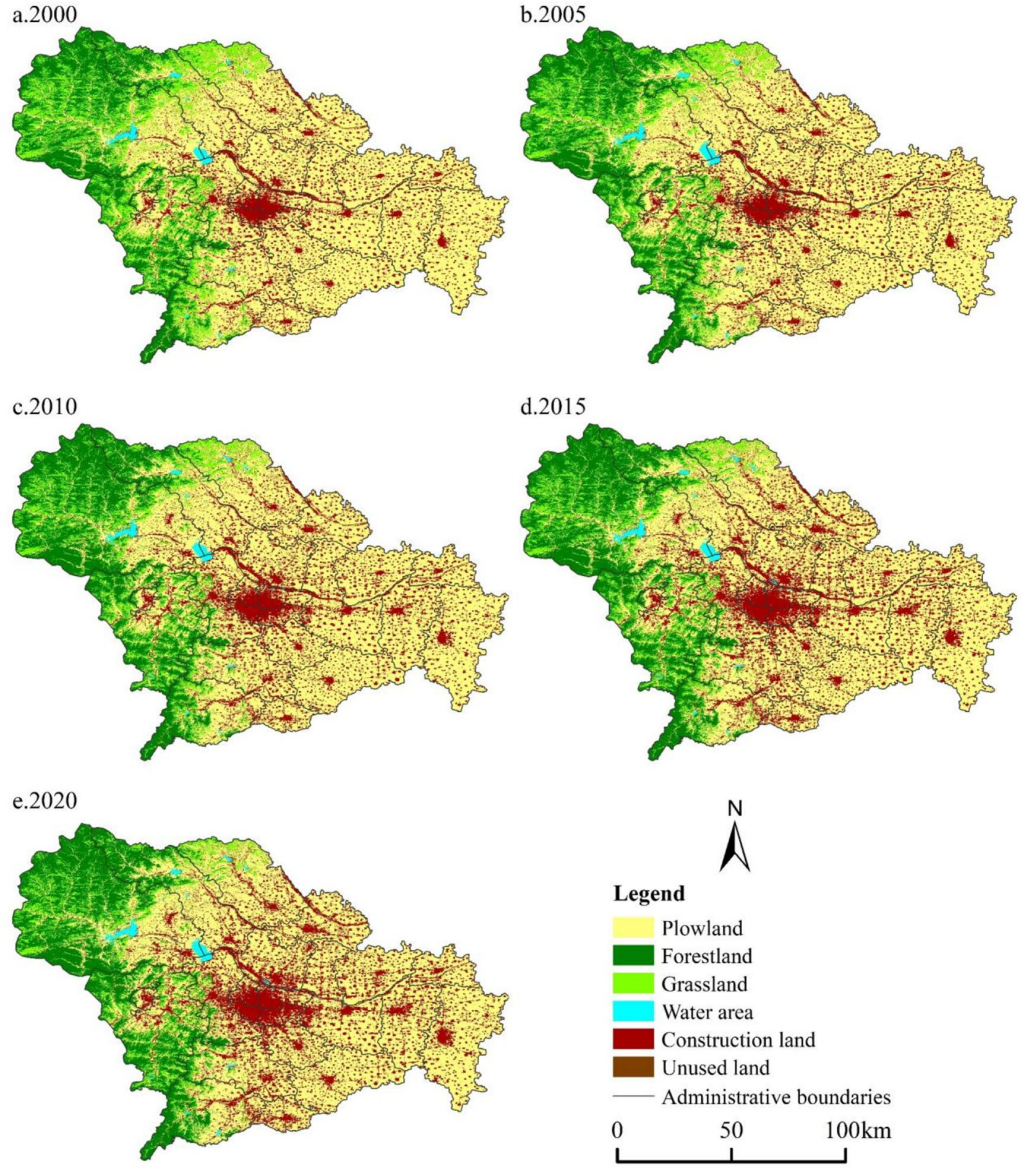
Land use spatial distribution in Shijiazhuang in 2000, 2005, 2010, 2015, and 2020

Plowland, forestland, and construction land are the main types of land in Shijiazhuang (Fig. 2). Plowland is concentrated in the east plain area, but with the continuous growth of urban construction demand for construction land, it is rapidly being converted into construction land, leading to a gradual decrease in plowland size and an increase in fragmentation. The western mountainous areas are the areas where forestland is concentrated, and the protection of forests and the vigorous promotion of the return of grain plots to forestry and the reclamation in mining areas make the distribution of forestland gradually increase in Shijiazhuang. The expansion trend of construction land on the edge of the city center is obvious, while the scale of construction land in other districts and counties is also increasing.

**Fig. 3:**
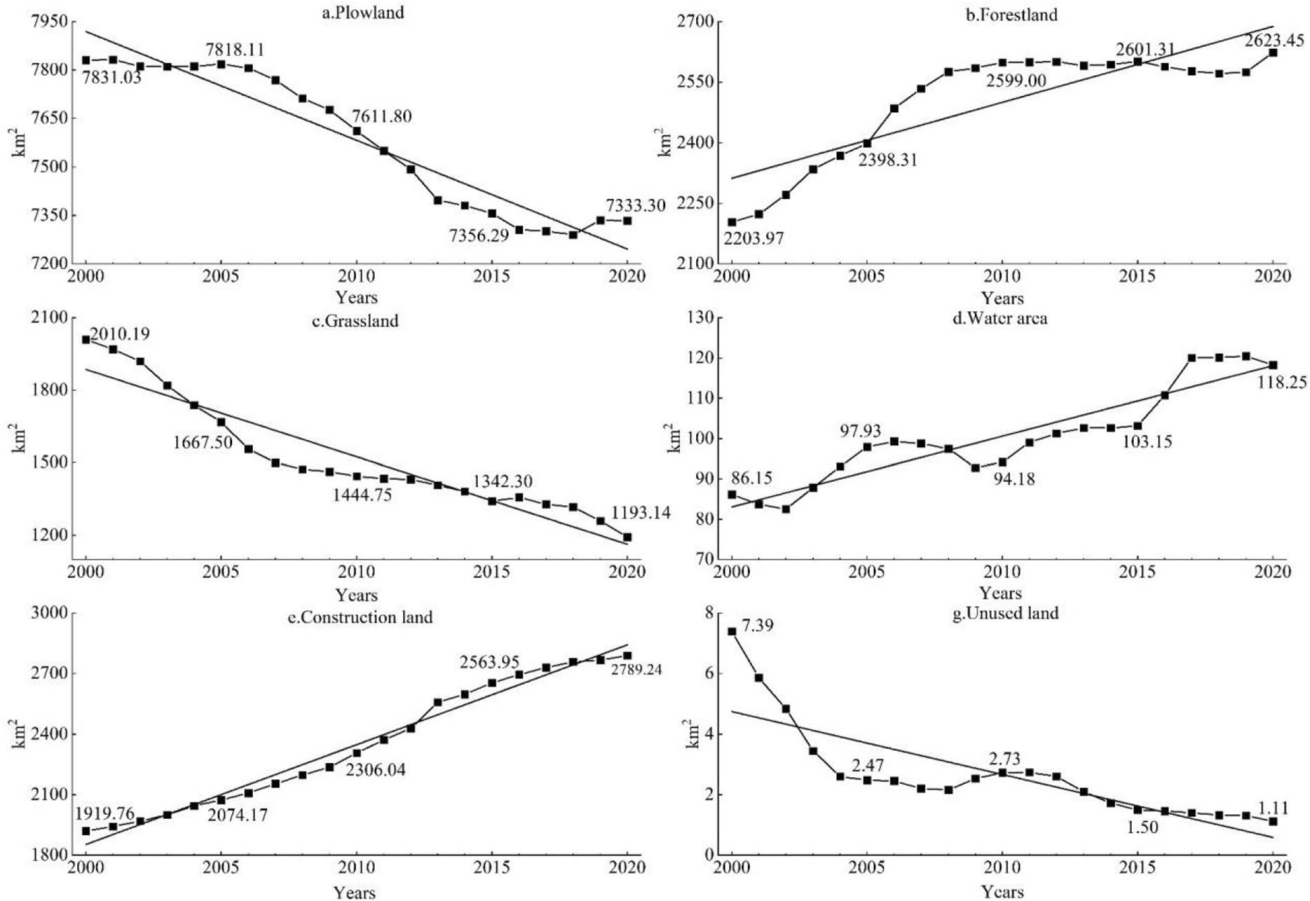
Area change of different types of land use in Shijiazhuang from 2000 to 2020

According to the change of land use area (Fig. 3), from 2000 to 2020, the areas of plowland, grassland, and unused land reduced by 497.73 km^2^, 817.05 km^2^, and 6.28 km^2^, while the areas of forestland, water area, and construction land increased by 419.48 km^2^, 32.10 km^2^, and 968.4 8km^2^. The plowland in Shijiazhuang shows a fluctuating downward trend, and in the early stage of the study, the urban construction in Shijiazhuang was mainly based on the transformation of the old city, and the plowland in the urban fringe remained relatively stable, with a slow decrease; however, with Shijiazhuang entering the stage of rapid urbanization, the demands for land for infrastructure construction, population resettlement, and industrial development continue to promote the expansion of construction land, and under the joint influence of ecological returning plowland, the reduction of polwland speeds up; thanks to the support of the land policy during the poverty alleviation period, Xingtang, Pingshan, Zanhuang, and other places supplemented cultivated land through land remediation and reclamation of land for construction land, and the reduction rate of cultivation land slowed down and even increased slightly. There is a high negative correlation between the changing trend of forestland and grassland. During 2000–2010, the ecological projects such as closed mountain forestation and ecological fallowing were implemented on the ground, the forestland maintained a rapid growth rate, and the green ecological barrier was basically formed in Taihang Mountain area in western Shijiazhuang; from 2010 to 2020, the natural evolution of vegetation and the construction of forest parks are the new kinetic energy for the slow growth of woodland. Land use allocation follows the principle of maximizing socio-economic benefits and the spatial distribution properties of forestland-grassland-plowland in Shijiazhuang, speeding up the conversion of grassland to forestland (to promote the maximization of ecological benefits) and grassland to plowland (to guarantee food security), making it the land type with the most serious area loss. As far as the water area is concerned, intermittent area fluctuations are influenced by climatic factors, while the ecological wetland construction and protection of the Hutuo River watershed are the decisive factors in the rise of the water area. The change of construction land area is closely related to urban development planning. As mentioned above, the early transformation of the old city makes the construction land increase at a slow rate, but the demand for urbanization land continues to increase, and the construction land area increases sharply, and the balance policy of plowland proportion in the post-poverty alleviation period slows down the growth momentum of construction land. Unused land is the predominant channel for the expansion of construction land and the supplement of plowland decreased by nearly two-thirds from 2000 to 2010, and then declined slowly.

### Variations in ecosystem service value

**Fig. 4:**
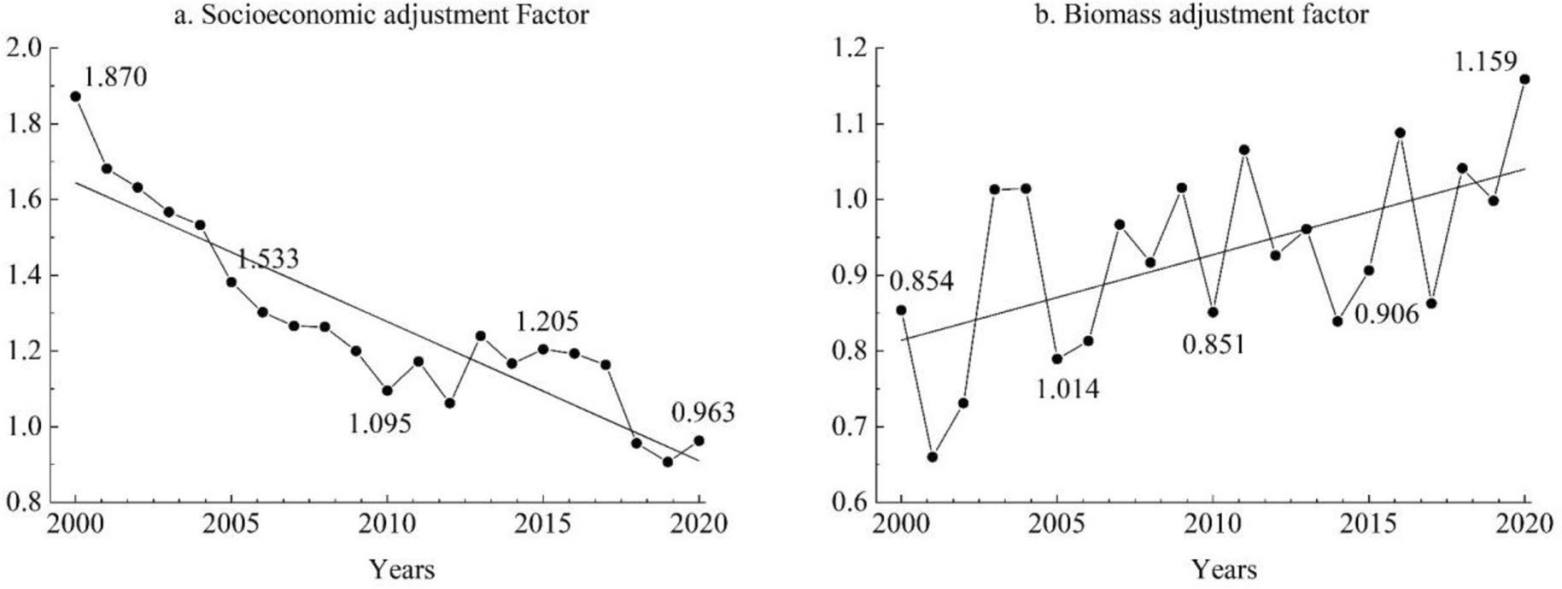
Parameters of dynamic evaluation model of ecosystem service value in Shijiazhuang from 2000 to 2020

Based on the dynamic evaluation model of ESV, we calculated the socio-economic adjustment coefficient and biomass factor adjustment coefficient from 2000 to 2020 (Fig. 4). The socio-economic adjustment coefficient showed an overall fluctuating downward trend, decreasing from 1.870 in 2000 to 0.963 in 2020. The biomass factor coefficient shows a fluctuating upward trend, rising from 0.854 in 2000 to 1.159 in 2020, and the gap between the NPP index and the national average NPP is gradually narrowing, even surpassing.

**Fig. 5:**
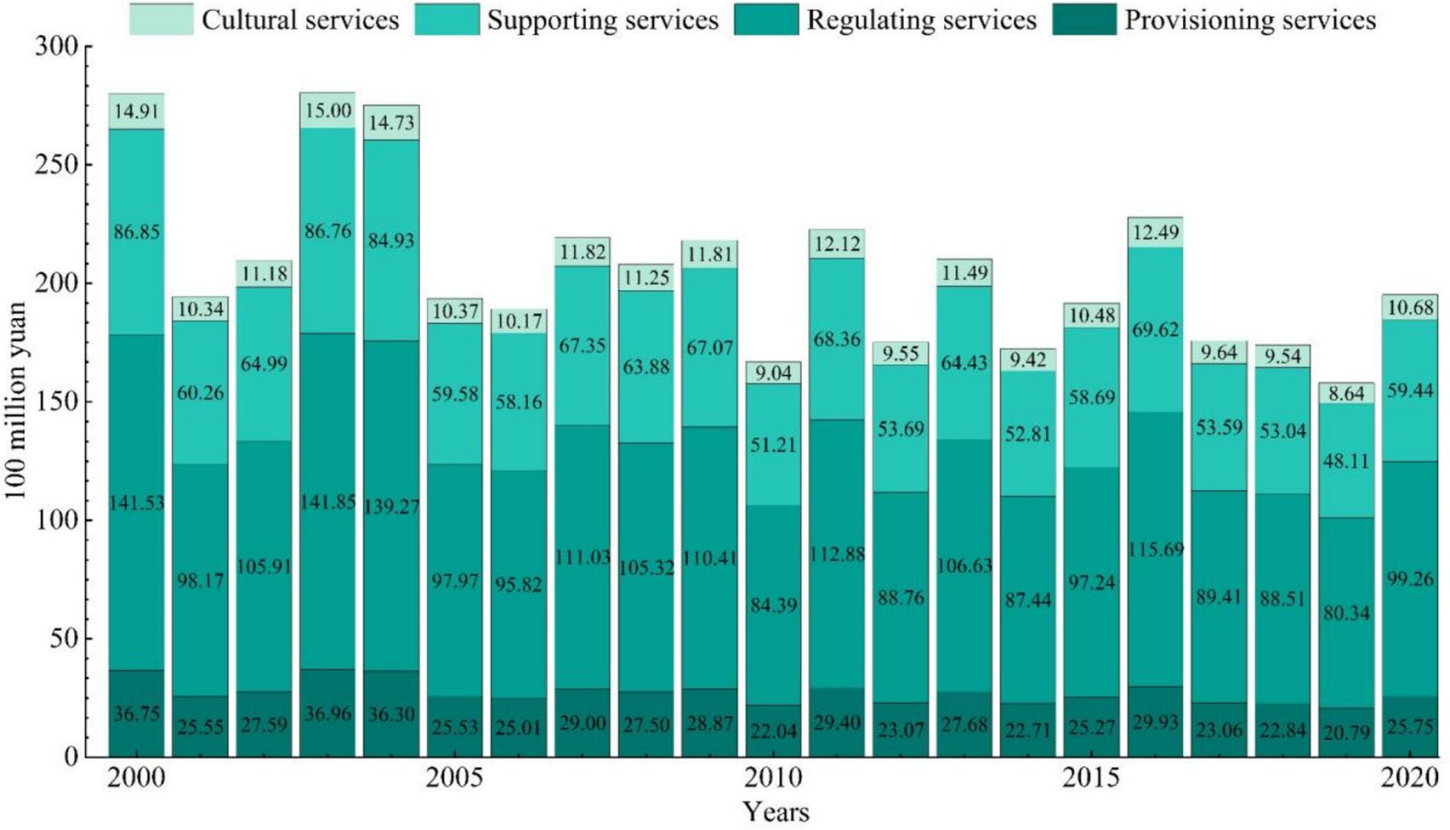
Ecosystem service value of Shijiazhuang from 2000 to 2020

After calculating the adjustment coefficient of the model, we calculated the ESV of Shijiazhuang between 2000 and 2020 (Fig. 5). From 2000 to 2020, although the fluctuation of socio-economic adjustment coefficient and biomass adjustment coefficient will further enlarge or narrow the annual difference of ESV, resulting in the fluctuation of ESV, land use change is the key factor that dominates the trend of ESV change in Shijiazhuang.

In 2001, the ESV of Shijiazhuang experienced its first drastic decline, with a decrease of 8.571 billion yuan compared to 2000, mainly due to the high temperature and low rainfall climatic conditions, which had a negative impact on ecosystem function and structure; between 2001 and 2003, the ESV gradually rebounded to 28.057 billion RMB, reaching the peak during the study period, thanks to the growth of water area and a reversal of climate conditions, with an increase in rainfall. However, between 2003 and 2006, due to the continuous decrease of grassland and unused land and the fluctuation of climate conditions, the ESV of Shijiazhuang shifted from an increase to a decline, experiencing its second drastic decline from 28.052 billion yuan to 18.916 billion yuan. Between 2006 to 2010, the ESV in Shijiazhuang fluctuated and decreased, with a total decrease of 2.248 billion yuan. From 2010 to 2020, due to human activities, land types with high ESV were transformed into construction land during urban development, laying the fundamental direction for the transformation of regional ESV. In addition, the social and economic development levels of Shijiazhuang gradually lagged the national average, further exacerbating the downward trend in ESV. In summary, the total value of ecosystem services in Shijiazhuang has shown a fluctuating downward trend, with the amplitude of fluctuations gradually decreasing, from 28.003 billion yuan in 2000 to 19.513 billion yuan in 2020, resulting in a loss of 8.49 billion yuan in ESV.

The trend of individual ESV is highly consistent with the overall ecosystem service value, showing a fluctuating downward trend. Among them, regulating services have an absolute advantage, but their value has decreased from 14.153 billion yuan in 2000 to 9.926 billion yuan in 2020, a decrease of 4.227 billion yuan, which is the most serious decline among individual ESV. The other three ESV have also declined to varying degrees, with provisioning services decreasing from 3.675 billion yuan to 2.575 billion yuan, a loss of 1.1 billion yuan, supporting services decreasing from 8.685 billion yuan to 5.944 billion yuan, a decrease of 3.741 billion yuan, and cultural services decreasing from 1.491 billion yuan to 1.068 billion yuan, a decrease of 0.423 billion yuan.

**Fig. 6:**
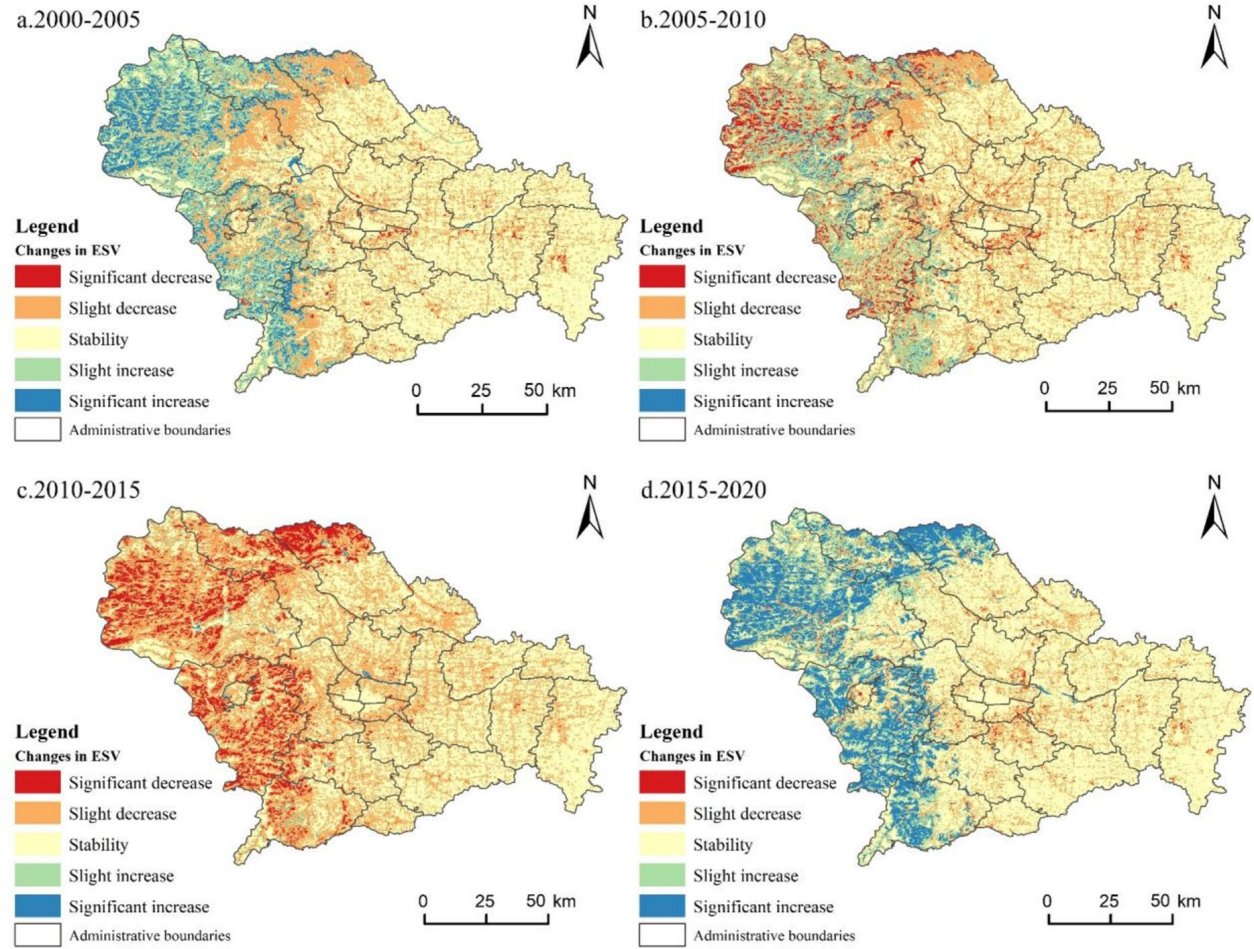
Spatial change of ecosystem service value in Shijiazhuang from 2000 to 2020

ESV is the spatial mapping of land use, and as such, we constructed a 300 × 300 m fishing net by leveraging the land use data to plot the spatial changes of ESV in four periods 2000–2005, 2005–2010, 2010–2015, and 2015–2020 to reflect the spatial change law of ESV (Fig. 6).

From 2000 to 2005, the areas of dramatic decline in the ESV were concentrated in the periphery of the existing built-up areas of the districts and counties. This is the result of the growth in the scale of construction land and the conversion of plowland to construction land. It is more significant in Xinhua District, Yuhua District, and Xinji City, which have rapid economic development. It is also worth noting that the intensity of land use development decreases as the distance from urban built-up areas increases, and the decline in the ESV moderates. The areas with increasing ESV are concentrated in the western mountainous areas, where the policies of mountain closure and ecological fallowing have significant effects, and the stability of regional ecosystems has been enhanced, with different increases in ESV. From 2005 to 2010, exploitation of forest resources and cultivation of cropland in the northwestern region increased disturbance to the ecosystem, and there was a dramatic decline in ESV. At the same time, the development of plowland along the Huanghetan reservoir and the urban construction of plowland have also caused a serious loss of ESV. The area of rising ESV is also concentrated in the western region, which is closely connected with the area of declining ESV, and it is the main area for the implementation of ecological restoration projects. From 2010 to 2015, the spatial changes in the ESV in Shijiazhuang were dramatic. Both western and northern mountainous areas experienced large-scale declines in ESV. This is closely related to the spatial restructuring of cropland, grassland, and forestland. In contrast, the distribution of ecosystem service rise areas is fragmented, and the percentage is relatively low. They are mostly concentrated in reservoir and river areas. From 2015 to 2020, there is a significant increase in ESV in the western mountainous regions, which is mainly influenced by the transition of arable land use. At the same time, the increase in biomass adjustment factor further amplifies the spatial increase in ESV. However, the negative impacts of continued urban growth on the ESV continue to intensify. Overall, the western mountainous region is a hotspot for changes in the ESV. It has experienced a dramatic fluctuation process of growth-decline-growth. The change in ESV in the plains is relatively homogeneous, and it revolves around urban expansion leading to varying degrees of decline in ESV.

**Fig. 7:**
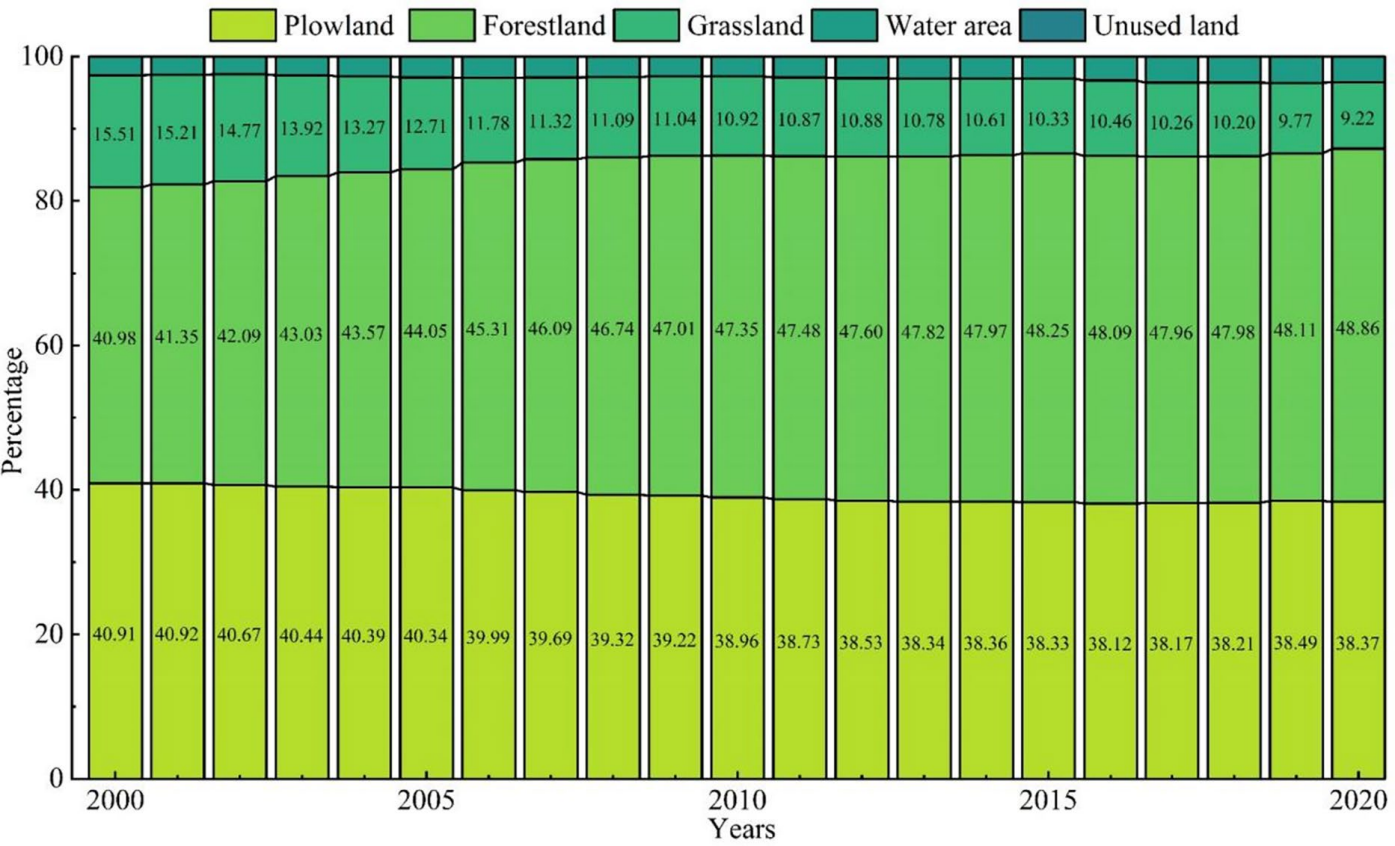
Contribution of ecosystem service value of distinct types of land use in Shijiazhuang from 2000 to 2020

Judging from the percentage of ESV in different regions (Fig. 7), plowland, forestland, and grassland provide over 90% of the total ESV. Although the water area is gradually increasing, the benefits of ESV are not significant, and the proportion of unused land ESV is extremely low. From 2000 to 2020, a series of ecological protection and construction policies promoted the expansion of the forest land area, further consolidated the dominant position of forestland ESV, and the contribution of forestland ESV increased from 40.98 to 48.86%. Due to the development and utilization of grassland resources, the contribution of grassland ecosystem services gradually decreased from 15.51 to 9.22%. The contribution of plowland ESV decreased gradually, by 2.54% in 21 years, but the contribution of plowland ecosystem service value remained at a prominent level. The contribution of ESV of water area and unused land is on the rise, from the initial 2.60 to 3.55%.

### Forecast of ecosystem service value

#### Forecast of land use

By leveraging the land use data of Shijiazhuang in 2015, we simulated the land use distribution of Shijiazhuang in 2020 with a time step of 5 years and compared it with the actual land use distribution data of 2020 (Table 3). In general, the area errors and error proportions of each land use type performed well, with the largest error area for grassland at 7.33 km^2^, but with a relatively low error proportion of only 0.61%; the area error of unused land accounts for the largest proportion, which is − 10.91%, but the total area of unused land is only 1.11 km^2^, so the quantity error is small. In space, the kappa coefficient is 0.96, indicating that the simulated results of land use in Shijiazhuang in 2020 were in good agreement with the actual results. To sum up, the CLUE-S model performed well in simulating the quantity and spatial distribution of land use in Shijiazhuang and can be further used for predicting land use in 2025.

**Table 3:** Comparison between simulation results and actual results of land use in Shijiazhuang in 2020

	Plowland	Forestland	Grassland	Water area	Construction land	Unused land
Actual area in 2020	7333.30	2623.45	1193.14	118.25	2789.24	1.11
Simulation area in 2020	7325.13	2621.92	1200.46	120.97	2789.01	1.00
Error area	− 8.17	− 1.54	7.33	2.72	− 0.23	− 0.11
Error percentage	− 0.11%	− 0.06%	0.61%	2.25%	− 0.01%	− 10.91%
Kappa	0.96					

Based on the conversion elasticity coefficient and spatial constraint of the CLUE-S modeling setting, the land use quantity for 5 consecutive years from 2021 to 2025 is simulated by using the land use data of Shijiazhuang in 2020 (Table 4). The land use change in the 5-year period continues the trend of land use change in Shijiazhuang during the historical period, with the area of plowland, grassland, and unused land have continued to decrease, with a decrease of 172.08 km^2^, 129.93 km^2^, and 0.33 km^2^. The areas of forest land, construction land, and water area have continued to grow, with the largest increase in construction land area, which increased by 172.74 km^2^ over the 5-year period, while the areas of forest land and water bodies increased by 112.38 km^2^ and 17.20 km^2^.

**Table 4:** Predicted land use area of Shijiazhuang from 2021 to 2025 (km^2^)

Years	Plowland	Forestland	Grassland	Water area	Construction land	Unused land
2020	7333.30	2623.45	1193.14	118.25	2789.24	1.11
2021	7298.51	2642.38	1174.67	120.81	2821.17	0.96
2022	7257.97	2665.83	1149.91	124.08	2859.80	0.91
2023	7224.76	2686.18	1123.96	127.83	2894.90	0.87
2024	7193.12	2711.84	1092.40	134.03	2926.27	0.84
2025	7161.24	2735.83	1063.21	135.45	2961.98	0.79

**Fig. 8:**
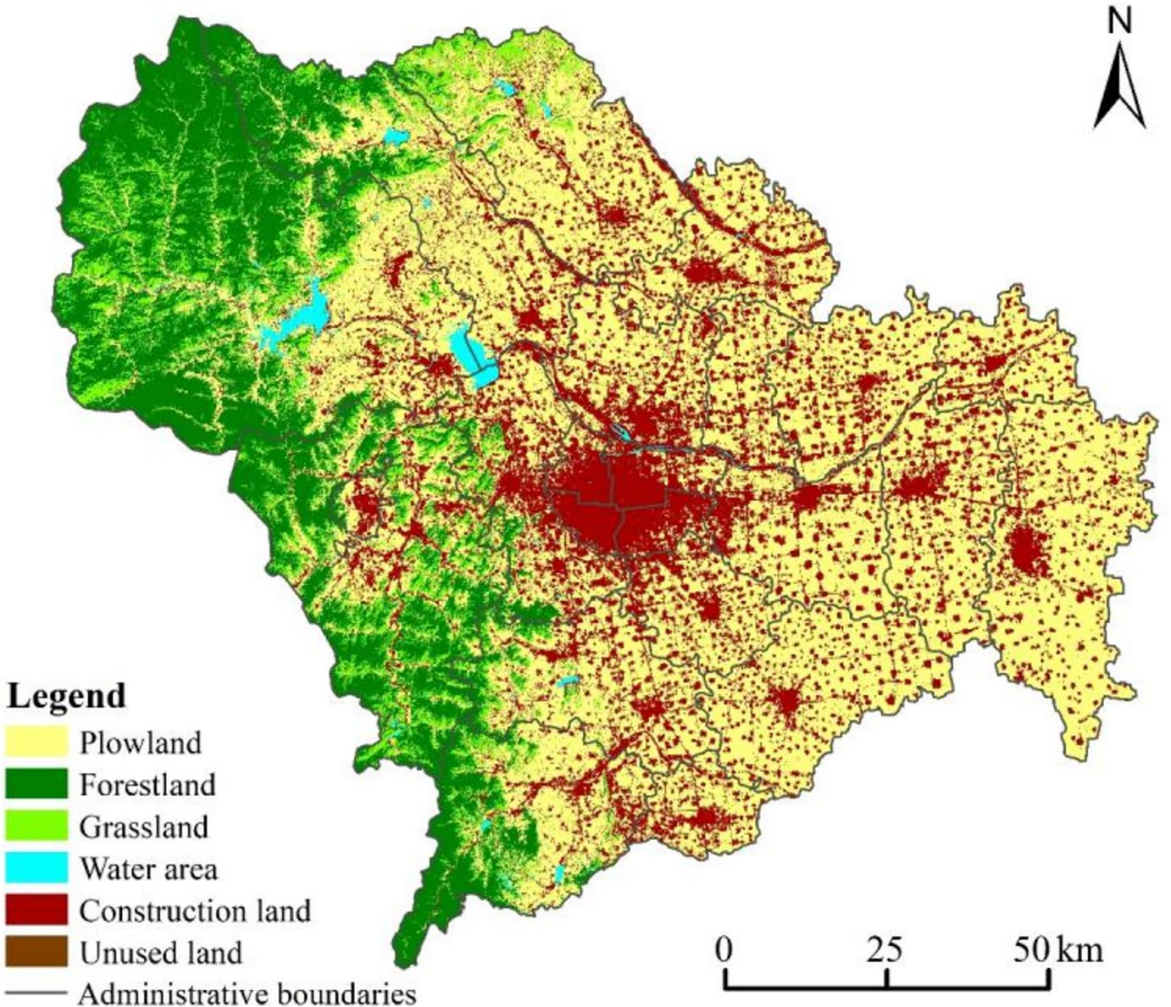
Prediction of the spatial distribution of land use in Shijiazhuang in 2025

The spatial change of plowland is concentrated in the western mountainous areas and around the already built-up areas of the districts and counties, which is the result of the combined effect of withdrawing from plowland for ecology policy and regional development and construction demands. The spatial agglomeration of forestland has increased, the forest coverage rate has improved, and ecological environmental protection has been effective. The pattern of grassland and water area is relatively stable with little change. The scale of construction land is further expanded, and the spatial agglomeration effect in the main urban area is obvious. The degree of development and utilization of unused land has increased, and the distribution is more fragmented (Fig. 8). In a comprehensive view, Shijiazhuang’s ecological land in 2025 shows an overall growth trend. The continuous growth of forestland and water area is conducive to building a firm ecological security barrier in the western mountainous areas, which is highly compatible with the systematic promotion of ecological space construction emphasized in the Shijiazhuang Territorial Spatial Master Plan (2021–2035). The growth of construction land is the result of the continuous urbanization process. The 14th Five-Year Plan of Shijiazhuang states that the city’s resident population is expected to reach 12 million by 2025 and that it is necessary to promote the development model of urban clusters and improve the comprehensive carrying capacity of the city. Benefiting from the most stringent plowland protection policy, the reduction has slowed down despite the continuous reduction of plowland.

#### Prediction of value correction factors for ecosystem services

Based on the gray forecast model, we forecast the GDP, Engel coefficient, and urban population ratio of the whole country and Shijiazhuang City from 2021 to 2025 (Table 5). From the perspective of model accuracy evaluation indicators *P* and *C*, all the prediction data meet the conditions of *P* > 0.95 and *C* < 0.35, the model accuracy level is excellent, the prediction results are reliable, and can be used for dynamic prediction of ecosystem service value.

**Table 5:** Prediction results of dynamic assessment parameters for ecosystem services value

Index	Years					Accuracy test	
	2021	2022	2023	2024	2025	$P$	$C$
National per capita GDP	78,040.20	83,152.58	88,439.16	93,905.86	99,558.84	1	0.005
Engel’s coefficient of national towns	27.20	26.78	26.37	25.96	25.56	1	0.041
National rural Engel coefficient	28.95	28.26	27.59	26.94	26.30	1	0.031
National urbanization rate	65.30	67.01	68.77	70.57	72.42	1	0.007
Engel’s coefficient of Shijiazhuang Town	24.33	23.60	22.87	22.16	21.45	1	0.253
Shijiazhuang rural Engel coefficient	22.69	21.94	21.18	20.44	19.70	1	0.094
Shijiazhuang urbanization rate	70.81	73.11	75.49	77.93	80.46	1	0.005
Shijiazhuang’s per capita GDP	67,597.90	71,213.80	74,932.46	78,756.80	82,689.82	1	0.054

Combining the projections with future climate scenario data for SSPs119, we calculated biomass adjustment factors and socio-economic adjustment factors for the projected period of 2020–2025 (Table 6). As the gap between the national per capita GDP and the per capita GDP indicators of Shijiazhuang increases, the willingness to pay coefficient will continue to decline; meanwhile, the proportion of the urban population and the gap between urban and rural Engel coefficients will also decrease, and the adjustment coefficient of economic and social development in the dynamic assessment of the ESV will continue to decline. The biomass factor coefficient reflects regional climatic differences, compared with 1.159 in 2020, slightly decreased between 2021 and 2025, with fluctuations in the short term.

**Table 6:** Dynamic assessment adjustment factors for the value of ecosystem services, 2021–2025

Category	2021	2022	2023	2024	2025
$At$	1.039	0.979	1.044	0.975	0.992
$PI$	0.994	0.985	0.977	0.968	0.960

#### Value prediction of ecosystem services

**Fig. 9:**
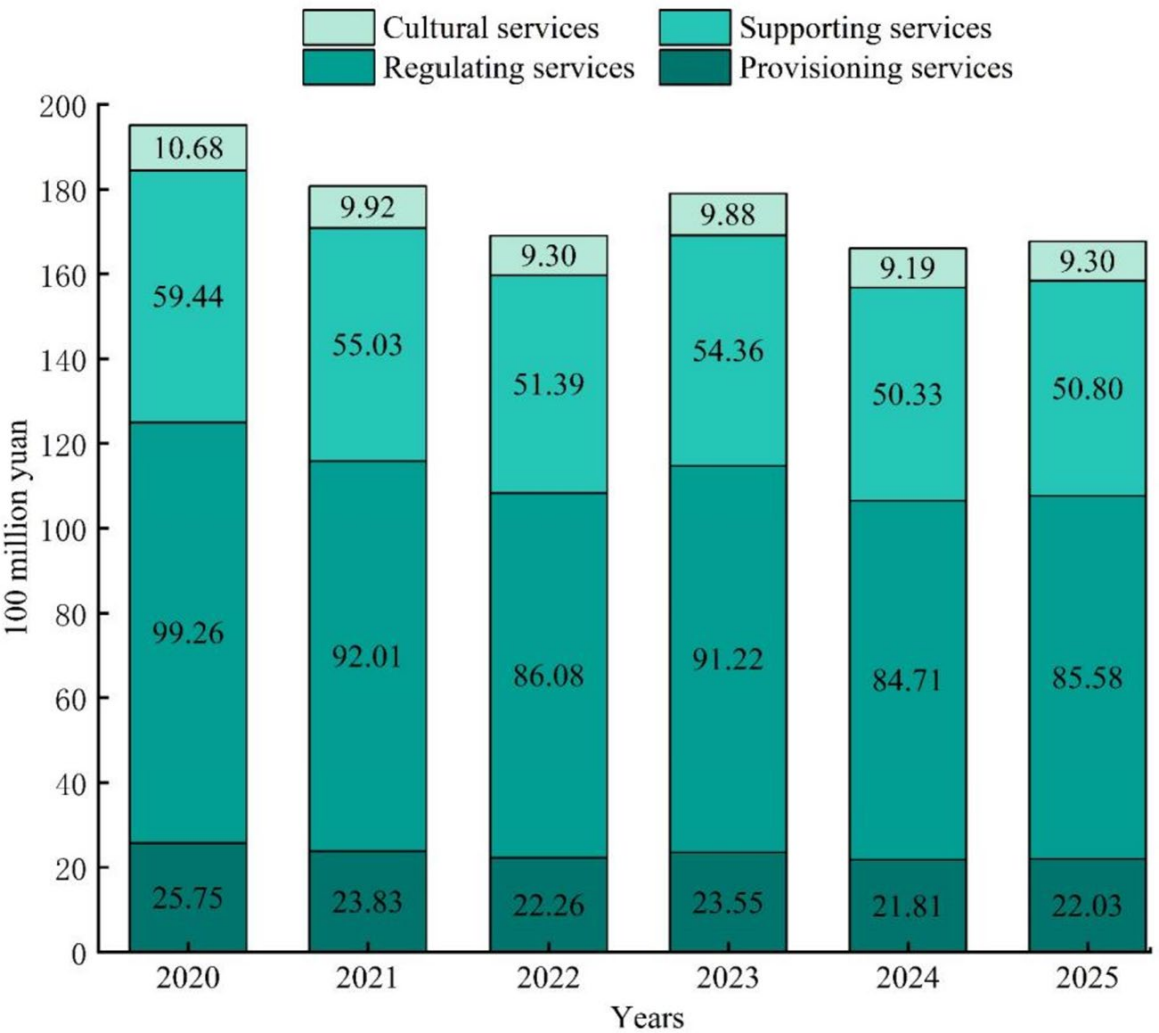
Ecosystem services value in Shijiazhuang from 2021 to 2025

Combining land use forecasts and ecosystem service correction factor predictions, the ESV in Shijiazhuang from 2021 to 2025 was further calculated (Fig. 9). Between 2020 and 2025, the fluctuation of the adjustment parameters of the dynamic assessment model of ESV weakened (Table 6), and the fluctuation range of ESV decreased, from 19.513 to 16.771 billion yuan between 2020 and 2025, the ESV was lost by 2.742 billion yuan, and the average annual loss of ESV was 457 million yuan. Among the individual ESV, the regulating services declined the most, at 1.455 billion yuan, and the loss of the remaining three services was 394 million yuan in provisioning services, 911 million yuan in supporting services, and 149 million yuan in cultural services.

## Discussion

### Dynamic assessment of ESV

ESV is the monetized expression of ecosystem services in a market economy (Xie et al., 2008). Conducting ESV assessments can improve society’s awareness of ecosystem services and help managers to develop appropriate ecosystem service management strategies (Yang et al., 2020; Zhang et al., 2020a). Urban ESV valuation, as an important category of ESV valuation, can reveal the trends and patterns of urban ESV under the background of increasing socio-economic levels and intensity of human activities. This topic has received wide attention and has resulted in a series of corresponding research results (Liu et al., 2021a, b; Yu et al., 2022). However, most studies lack the exploration of the spatial heterogeneity of ESV (Chen et al., 2021; Zhang et al., 2020b, 2022), but the monetary estimation of ESV itself is influenced by factors such as market prices, willingness to pay, and resource scarcity (Fu et al., 2022; Su et al., 2020). Therefore, the study is based on the equivalence factor method, using socio-economic and biomass factor adjustment coefficients to correct to for regional differences and stage characteristics in ESV assessment, thereby improving the accuracy of ESV results.

Furthermore, compared with time-section ESV assessment (Cai et al., 2022; Su et al., 2020), continuous time-series ESV assessment offers a comprehensive demonstration of the data characteristics of single-year ESV (Wang et al., 2021). It reveals the meticulous and objective fluctuating change process of ESV and supports the establishment of a long-term ecosystem service monitoring network (Jin et al., 2021; Yu et al., 2018). In previous studies (Fu et al., 2022; Su et al., 2020; Yuan et al., 2019a, b), socio-economic and biomass adjustment coefficients were often averaged over multiple years, which weakened the spatial and temporal dynamic characteristics of ESV. To address this limitation, we determined the adjustment coefficients for each year based on the state of socio-economic development and the climatic and environmental characteristics specific to each year. Additionally, we combined this information with the land use data of each year to construct a dynamic assessment model of ESV, showcasing year-to-year differences in ESV. These differences can even surpass the differences observed under different time cross-sections (Fig. 5). More detailed data can help recognize and understand the spatial and temporal non-smoothness of ESV (Li et al., 2021a; Wang et al., 2021).

In the ESV prediction stage, we coupled CLUE-S and GM (1,1) models to explore the trend of ESV evolution in Shijiazhuang from 2021 to 2025, which fills the corresponding gap of existing studies that conduct ESV prediction only from the perspective of land use prediction (Gao et al., 2021; Xi et al., 2021; Zhang et al., 2022). Land use change is the main factor affecting the value of ESV (Zhang et al., 2020a, 2020b), but market conditions, climate characteristics, and economic level also affect ESV (Fu et al., 2022), and relying only on land use projection data to assess ESV is to be optimized. Based on the existing studies (Gao et al., 2021; Peng et al., 2021), we used the CLUE-S model for land use projections, which has a high accuracy of construction land simulation and can better simulate the urban development process of Shijiazhuang and provide a land use data basis for ecosystem service value projections; meanwhile, we used the GM(1,1) projection data to calculate the socio-economic adjustment coefficient for 2021–2025, and the biomass adjustment coefficients were calculated by the SSP119 scenario data. Combining the land use data and adjustment coefficients, we make ESV projections (Fig. 9), which highlight the spatial and temporal dynamic characteristics of ESV while ensuring the coherence of the time series.

### Policy construction

ESV plays a crucial role in assessing the stability of ecosystem structure and function, providing a valuable reference for regional ecological construction, ecological compensation, and policy formulation (Peng et al., 2021; Wei et al., 2022; Wu et al., 2020). Forestland has the most profound impact on the ESV in Shijiazhuang, highlighting the imperative need for attentive forest conservation and management. It is essential to augment ecological funding (Guo et al., 2021), adaptively nurture and refurbish forested areas, and proactively advance afforestation initiatives like converting cultivated land back to forests and promoting reforestation in the western mountainous regions. Amid the rapid urbanization, Shijiazhuang faces prominent issues of urban construction encroaching upon plowland. There is a pressing need to bolster the protection of plowland, striking a delicate balance between agricultural land conservation and economic growth. Enhancing the ecosystem service functions of plowland can be achieved through refining agricultural infrastructure and optimizing the agricultural landscape structure. The continuous growth in construction land warrants significant attention. It is crucial to emphasize regional land-use planning as the foundation for coordinated land-use layout, fostering guided and orderly growth of construction land. Vigorous efforts should be directed toward urban renewal, reclamation of abandoned industrial and mining lands, and other measures to diversify sources for construction land. Such actions will mitigate the adverse effects of the rapid expansion of construction areas on regional ecosystem services.

The level of regional socio-economic development provides valuable information regarding individual consumption intentions and preferences, serving as a significant basis for ESV assessment (Su et al., 2020). The continuous decline of the socio-economic adjustment coefficient is one of the factors contributing to the decrease in ESV. It reflects the regional willingness to pay and consumption preferences, highlighting the slower growth rate of Shijiazhuang’s socio-economic development compared to the national average and the evident lack of economic growth momentum. To address these challenges, it becomes crucial to promote iterative upgrading, cluster development, and integration and innovation within Shijiazhuang’s traditional industries. Breaking the barriers of administrative districts and industries, a comprehensive plan for the development of the urban economy, ecological economy, and industrial economy should be devised, leveraging regional resource endowment zoning to drive high-quality development and foster a new development pattern.

### Research limitations and outlook

The study of ESV contributes to the construction of regional sustainable development (Wang & Cao, 2021a, b). However, the study has some limitations in terms of the model mechanism. Firstly, the accuracy and resolution of land use data have an impact on the precision of ecosystem service valuation to some extent (Liu et al., 2020). Although the overall accuracy of the land use data used in the study is high, the classification accuracy of built-up land is relatively low. This implies that land types such as plowland, forestland, and grassland may be misclassified as built-up land. Additionally, due to the limitations of the CLUE-S model’s data processing, the resolution of the land use data had to be adjusted to 100 × 100 m, which restricts the ability to capture detailed internal characteristics of the land use system. Secondly, the ESV encounters uncertainties due to the complex, dynamic, and nonlinear nature of ecosystems (Shoyama et al., 2017; Zhang et al., 2022). In this study, the introduction of socioeconomic and climatic condition factors for regional adjustment of ESV represents a positive step. In future studies, it is recommended to consider land use mapping based on high-resolution remote sensing imagery to conduct ESV. This approach can help reveal the ESV of urban green areas that are often overlooked within built-up land and have significant implications for urban residents’ well-being, as well as urban management and construction (Chen et al., 2022; Hou et al., 2021). Furthermore, incorporating field surveys can enhance the accuracy of land use classification results and improve the reliability of ESV assessments. These surveys contribute to refining the classification of land use types. Moreover, it is crucial to develop complex nonlinear spatial models that quantitatively assess the impacts of climate, economic, and policy factors on ESV. Such models can provide a deeper understanding of the relationships and interactions between these factors (Li et al., 2022; Shoyama et al., 2017). By exploring and implementing advanced modeling techniques, improvements can be made to the existing methods used in ecosystem service valuation.

This study comprehensively explores the temporal and spatial fluctuation characteristics of ESV through dynamic assessment and prediction. It utilizes detailed data to demonstrate that the ESV does not follow a stable pattern of increase or decline. Moreover, it verifies the impact of changes in the natural environment and socio-economic development on the value of regional ecosystem services, which serves as the objective cause for the dynamic fluctuations in ESV. These impacts should be taken into account in the assessment of ESV. The study emphasizes that while land use is closely linked to the ESV, it is also influenced by natural conditions and socio-economic development. Therefore, the assessment of ESV should consider multiple factors that affect their value, in order to achieve accurate assessments. Management and regulatory measures for ESV should account for their fluctuations and develop appropriate strategies based on long-term trends. Prediction of ESV is crucial for conservation and early warning purposes. However, such predictions should not be limited to land use projections; instead, they should provide more comprehensive and accurate data to consider the development trends influenced by various factors.

## Conclusion

In this study, based on the analysis of spatial and temporal changes in land use dynamics in continuous time series, the equivalent factor method was used to measure the ESV of Shijiazhuang City from 2000 to 2020 by constructing a dynamic valuation model of ESV, and to predict the ecosystem service value from 2021 to 2025 by using CLUE-S and CM (1,1) models.

By introducing dynamic socio-economic adjustment coefficient and biomass adjustment coefficient equivalent factor method, it can reveal the spatial and temporal non-equilibrium of ESV while reflecting regional spatial heterogeneity. The 2000–2020 ESV in Shijiazhuang showed a fluctuating downward trend, and the total ecosystem service values decreased from RMB 28.003 billion in 2000 to 19.513 billion yuan in 2020, of which the expansion of urban construction land is the main factor for the decline of ESV, but the fluctuating change process is the result of the influence of adjustment coefficients. In the case of Shijiazhuang, plowland, forestland, and grassland contribute more than 90% of the ESV, and together determine the trend of regional ESV change. The spatial variation of ESV in Shijiazhuang is characterized by obvious differentiation, and although the western mountainous areas have higher ESV, land use restructuring causes drastic fluctuations in ESV, and ecosystem stability faces severe adjustments. The prediction of ESV using CLUE-S and CM (1,1) models performs well and can reflect the spatial and temporal dynamics of ecosystem services, which has stronger reference significance.

The study of long time series analysis and prediction of urban ESV with dynamic assessment perspective can provide detailed reference basis for regional ecological protection, environmental monitoring, and management decision. However, how to further improve the reliability and scientific validity of ESV assessment still needs to rely on a high-precision data base and deepen the understanding of ecosystem service processes.

## Data Availability

The datasets generated during and analyzed during the current study are available from the corresponding author on reasonable request.
